# Hours spent and energy expended in physical activity domains: Results from *The Tomorrow Project *cohort in Alberta, Canada

**DOI:** 10.1186/1479-5868-8-110

**Published:** 2011-10-10

**Authors:** Ilona Csizmadi, Geraldine Lo Siou, Christine M Friedenreich, Neville Owen, Paula J Robson

**Affiliations:** 1 Department of Population Health Research, Alberta Health Services-Cancer Care 1331-29 Street NW, Calgary, Alberta, T2N 4N2, Canada; 2 Department of Population Health Research, Alberta Health Services - Cancer Care c/o Holy Cross Site, Box ACB, 2210 2nd Street SW, Calgary, AB, T2S 3C3, Canada; 3 National Health and Medical Research Council Senior Principal Research Fellow Head, Behavioural Epidemiology, Baker IDI Heart and Diabetes Institute, Level 4, 99 Commercial Rd, Melbourne, VIC 3004, Australia; 4 Department of Population Health Research, Alberta Health Services - Cancer Care Suite 1400, Sun Life Place, 10123 99th Street NW, Edmonton, AB, T5J 3H1, Canada

**Keywords:** physical activity, energy expenditure, sedentary behaviour, Canada, occupation, leisure-time, transportation

## Abstract

**Background:**

Knowledge of adult activity patterns across domains of physical activity is essential for the planning of population-based strategies that will increase overall energy expenditure and reduce the risk of obesity and related chronic diseases. We describe domain-specific hours of activity and energy expended among participants in a prospective cohort in Alberta, Canada.

**Methods:**

The *Past Year Total Physical Activity Questionnaire *was completed by 15,591 *Tomorrow Project*^® ^participants, between 2001 and 2005 detailing physical activity type, duration, frequency and intensity. Domain-specific hours of activity and activity-related energy expenditure, expressed as a percent of total energy expenditure (TEE) (Mean (SD); Median (IQR)) are reported across *inactive *(<1.4), *low active *(1.4 to 1.59), *active *(1.6 to 1.89) and *very active *(≥ 1.9) Physical Activity Level (PAL = TEE:REE) categories.

**Results:**

In *very active *women and amongst all men except those classified as *inactive*, activity-related energy expenditure comprised primarily occupational activity. Amongst *inactive *men and women in *active, low active *and *inactive *groups, activity-related energy expenditure from household activity was comparable to, or exceeded that for occupational activity. Leisure-time activity-related energy expenditure decreased with decreasing PAL categories; however, even amongst the most active men and women it accounted for less than 10 percent of TEE. When stratified by employment status, leisure-time activity-related energy expenditure was greatest for retired men [mean (SD): 10.8 (8.5) percent of TEE], compared with those who were fully employed, employed part-time or not employed. Transportation-related activity was negligible across all categories of PAL and employment status.

**Conclusion:**

For the *inactive *portion of this population, active non-leisure activities, specifically in the transportation and occupational domains, need to be considered for inclusion in daily routines as a means of increasing population-wide activity levels. Environmental and policy changes to promote active transport and workplace initiatives could increase overall daily energy expenditure through reducing prolonged sitting time.

## Introduction

The health benefits of physical activity are well known [1,2]. However, despite the widespread promotion of physical activity guidelines [3-5], it is apparent that a large proportion of the general population may not be sufficiently active to derive these benefits. In Canada, evidence suggests that leisure-time activity is increasing over time [6-9], but other aspects of daily life may be becoming increasingly more sedentary, potentially resulting in a net reduction in total energy expenditure (TEE) [10,11].

Historically, physical activity recommendations have focused on discretionary activity in leisure time [12], with the assumption that individuals have more flexibility and control over activity in this domain than in other domains such as occupation or transport. Surveys and epidemiologic studies have also focused on leisure-time activity, often encouraged by evidence that demonstrates a strong link between moderate and high intensity levels of leisure-time activity and cardiovascular fitness [13]. In addition, since leisure-time activities of moderate to high intensity can be associated with higher levels of energy expenditure, weight maintenance is assumed to be more achievable when leisure-time activities of higher intensity are performed on a regular basis [14]. Despite widely publicized recommendations and some apparent increases in the number of adults successfully meeting leisure-time physical activity guidelines, the prevalence of obesity and obesity-related chronic diseases continue to increase [11,15,16]. This trend has prompted an interest in the assessment of activity and energy expenditure in all domains, which may be amenable to differentiated and more-focused programs and policy initiatives [11,17-19]. Importantly, the study of those activities that comprise larger portions of the day is beginning to generate evidence suggesting that important health benefits may be gained by increasing activity in all domains [20,21].

Here we report findings on adult participation in leisure-time, occupation, household and transportation-related activity among a geographically dispersed population of Canadian men and women participating in the *Tomorrow Project*^®^, an Alberta province-wide cohort, designed to investigate the associations between lifestyle factors and chronic disease risk. Our objectives are to describe variations in hours spent and energy expended in domain-specific activities and to examine differences between domains at higher levels of physical activity with those at lower levels using a recognized criteria of physical activity level.

## Methods

### Study design and participants

The *Tomorrow Project*^® ^is a prospective cohort of Albertans established in 2001 to study the associations between various lifestyle factors and chronic disease outcomes. The recruitment methods for the *Tomorrow Project*^® ^have been described elsewhere [22]. Briefly, random digit dialing was used to recruit men and women between 35 and 69 years of age who had not been diagnosed with cancer, other than non-melanoma skin cancer. At baseline participants completed a health and lifestyle questionnaire, and the self-administered *Past Year Total Physical Activity Questionnaire *(PYTPAQ) [23]. Participants also provided information on employment status (full, part-time, not employed/homemaker/student/other or retired), education, marital status and household income. A total of 18,443 enrolled between February 2001 and January 2005 were eligible for this analysis. Excluded were those who did not complete the PYTPAQ (n = 2,405), pregnant women (n = 31), those recruited as 'second in household' (n = 344), those with prior history of cancer diagnosis (n = 33) and those with missing components in the PYTPAQ data (n = 39). Participants with missing PYTPAQs, and height and weight data (n = 2,444) were more likely to be male, slightly younger, and be employed full-time, however, education levels were similar to the rest of the study sample. The remaining excluded participants did not meet eligibility criteria to participate in the cohort. Ethical approval for baseline data collection in the *Tomorrow Project *was obtained from the Research Ethics Committees of the Alberta Cancer Board (now the Alberta Cancer Research Ethics Committee at Alberta Health Services) and the University of Calgary, Alberta, Canada.

### Time spent and energy expended in activities

The accelerometer-validated PYTPAQ [23] completed by cohort participants at the time of enrollment was the source of self-reported activity. The PYTPAQ has an open format table design that queries about employment and volunteer, recreation and leisure, household and do-it-yourself and transportation-related activities during the previous 12 months. Examples of physical activities within each domain were provided on the questionnaire in order to assist respondents in reporting their activities. Participants were asked not to include activities done while sitting in the recreation and leisure (e.g. playing cards and reading) and household sections of the PYTPAQ since the questionnaire was designed to capture activity; however, a full range of activities, including sitting, were ascertained in the employment and volunteer activity section since it was felt that it would be easier for participants to report a full range of occupational activities rather than just those that were performed seated. Participants were asked to describe activities and to report the frequency (months/year, days/week, hours or minutes/day) and perceived intensity of activities performed. Definitions of levels of intensity (1 = inactive (mainly sitting); 2 = light (mainly standing); 3 = moderate (slight increase in heart rate and some light sweating); and 4 = heavy (substantial increase in heart rate and heavy sweating)) were provided in the questionnaire, along with examples.

The frequency and duration of time reported for occupation (paid employment and unpaid volunteer work), recreation and leisure-time, household and transportation-related activities were used to estimate the hours of activity contributed by each domain and total hours of daily activity.

Descriptions of activities and self-reported intensities on the PYTPAQ were used to identify and assign appropriate *metabolic equivalents of task *(METs) using values published in the *Compendium of Physical Activities *[24,25]. First a standard MET value was applied to each reported activity that was derived from the Compendium and a self-reported intensity of activity was also recorded by the participants based on standard descriptions provided to them within the questionnaires. These self-reported intensity values were used to adjust the intensity assigned to each reported activity that was derived from the Compendium. Hence, for example, if a participant reported 'running', an average MET value for running would be used from the Compendium that could then be adjusted up or downward depending on the intensity level reported by the participant.

If the participant reported that the activity was 'vigorous', then, a higher MET value was assigned than if it was reported as 'moderate' or ' low'. In so doing, we were able to standardize the intensity values for different reported recreational activities but individualize them as well to reflect the actual energy expended by the participant.

For occupation we ascertained job titles as well as up to three descriptors of the type of physical activity that was done. Since the focus of this questionnaire was to capture the physical activity energy expenditure by type of activity, we used the job titles and descriptors of activity as a means of identifying the appropriate activity energy expenditure for each reported occupation rather than as a means of classifying the study population. Hence, we have very detailed data on occupational activity that was used in this analysis and not just employment status.

The hours per week reported for each activity were multiplied by the METs assigned to the activity. MET-hours per week and MET-hours per day were then determined for each domain (i.e., occupation, household, leisure-time and transportation). Total MET-hours per day was estimated by summing the MET-hours from each domain of activity. MET-hours per day were multiplied by kilograms of body weight to estimate the amount of energy expended, expressed in kilocalories, while engaging in each type of activity (1 MET = 1 kcal/kg/hour). In addition, the time spent in sedentary (1.5 METs and lower), light (>1.5 and <3 METs) and moderate to vigorous activities (3 METs or more) within each domain was also determined.

### Total energy expenditure (TEE)

Individual level activity-related energy expenditure, expressed in kilocalories, was estimated by summing the energy expended in all types of activity (described above). One MET was subtracted from each hour of active time to eliminate double counting of energy expenditure equivalent to resting energy expenditure (REE) for that time period. TEE was estimated using the following equation:

TEE=[[REE-(total hours/d of activity×weight (kg))]+[MET-hours/d×weight (kg)]]1.1

The sum of REE (estimated by the Schofield equation [26]) and activity-related energy expenditure was multiplied by 1.1 to account for the energy expenditure of the thermic effect of food [27].

Height and weight for REE estimation were self-reported by participants. A 183 cm (72 inch) tape-measure was mailed to participants along with detailed instructions for height measurement and weight measurement. Participants were asked to use a scale that was accessible to them. Follow-up by telephone was conducted by Tomorrow Project staff to clarify measurements that were not considered plausible.

### Physical activity levels (PAL)

The ratio of total energy expenditure to resting energy expenditure (TEE:REE) referred to as PAL was used to classify activity into four categories as described in *Dietary Reference Intakes for Energy, Carbohydrate, fiber*, *Fat, Fatty Acids, Cholesterol, Protein and Amino Acids *(Institute of Medicine of the National Academies), 2002 [27]: *inactive *(<1.40), *low active *(1.40 to 1.59), *active *(1.6 to 1.89) and *very active *(≥ 1.90).

### Domain-specific hours and activity-related energy expenditure

For each participant, we estimated daily number of hours spent and energy expended in kilocalories (means, standard deviations [SD], medians and interquartile ranges [IQR]) within each domain: occupation, leisure-time, household and transportation. Domain-specific hours (mean (SD)) were estimated by gender and PAL. The time spent in sedentary, light and moderate-to-vigorous activities within each domain, by PAL and by gender was also determined. In addition, total and domain-specific activity-related energy expenditure expressed as a proportion of TEE (mean (SD)) across PAL and employment status categories are reported by gender.

### Data analyses

Monotonic trends between daily active time or activity-related energy expenditure and activity levels were assessed by using the Jonckheere-Terpstra trend tests. Differences in medians or distribution shapes of the daily activity-related energy expenditure between activity levels or employment status were compared by using Kruskal-Wallis rank sum tests. All descriptive statistics (Means [SD], medians and IQRs) and analyses were performed using PROC NPAR1WAY (Kruskal-Wallis rank sum tests) and PROC FREQ (Jonckheere-Terpstra trend tests), available in the SAS/STAT software (version 9.1.3 of the Statistical Analysis System (SAS) for Linux. Copyright © 2005 SAS Institute Inc., Cary, NC, USA).

## Results

### Participant characteristics

The study population consisted of 6,134 men and 9,457 women, 35 to 69 years of age, enrolled in the *Tomorrow Project*^® ^between 2001 and 2005 (Table 1). Estimated mean REE and TEE were higher for men compared with women, but average PALs were comparable with the majority of men (49%) and women (43%) classified as *very active*. Overall participants reported around 8 hours of total daily physical activity, comprising primarily occupational activity in men, and occupational and household activity in women (Table 1).

**Table 1:** Socio-demographic, lifestyle, physical activity and energy-expenditure attributes of participants in the Tomorrow Project^a^.

	Men (N = 6,134)	Women (N = 9,457)
**Socio-demographic, lifestyle**		
Age (mean [SD])	50.5 [9.1]	50.6 [9.2]
BMI (mean [SD])	28.0 [4.3]	27.0 [5.8]
Education^b ^(% >high school)	74	69
Household annual income^c ^(% 60,000)	61	48
Marital status (% with partner)	82	74
Employment status^d^		
Full-time (%)	75	44
Part-time (%)	6	23
Not employed/homemaker/student/other (%)	6	20
Retired (%)	12	14
Current non-smoker^e ^(%)	80	81
**Daily number of hours and MET-hours of domain-specific activity (mean [SD])**		
Total activity (hrs/d)	8.1 [2.8]	7.9 [2.9]
Total activity (MET hrs/d)	25.3 [11.1]	22.9 [9.8]
Leisure (hrs/d)	0.9 [0.8]	0.8 [0.7]
Leisure (MET hrs/d)	4.2 [4.0]	3.5 [3.4]
Occupational time (hrs/d)	5.6 [2.7]	3.7 [2.6]
Occupational (MET hrs/d)	16.4 [10.5]	9.8 [8.0]
Household activity (hrs/d)	1.6 [1.3]	3.4 [2.1]
Household activity (MET hrs/d)	4.6 [4.0]	9.5 [6.3]
Transportation activity (hrs/d)	< 0.1	<0.1
Transportation activity (MET hrs/d)	0.2 [0.7]	0.1 [0.4]
**Daily energy-expenditure (mean [SD])**		
Resting energy expenditure (REE) (kcal/d)	1876 [195]	1438 [142]
Total energy expenditure (TEE) (kcal/d)	3726 [1013]	2766 [716]
Physical Activity Level (TEE:REE)	2.0 [0.5]	1.9 [0.4]

^a ^Includes people recruited in the first six recruitment waves (February 2001-January 2005) but excludes people who did not return a PYTPAQ (n = 2,405), pregnant women (n = 31), people who were recruited as 'second in household' in the first recruitment wave (n = 344), people with prior history of cancer (n = 33), and people with missing data on body weight or height (n = 39).

^b ^Education: 1 woman had missing data.

^c ^Household annual income (CAD): 78 men and 269 women had missing data. ^d ^Employment status: 3 men and 5 women had missing data.

^e ^Current non-smoker: those who previously smoked either daily or occasionally and were non-smokers at the time of completing baseline questionnaires; and those who never smoked in life. 1 woman had missing data.

### Time spent in domain-specific activities

**Figure 1:**
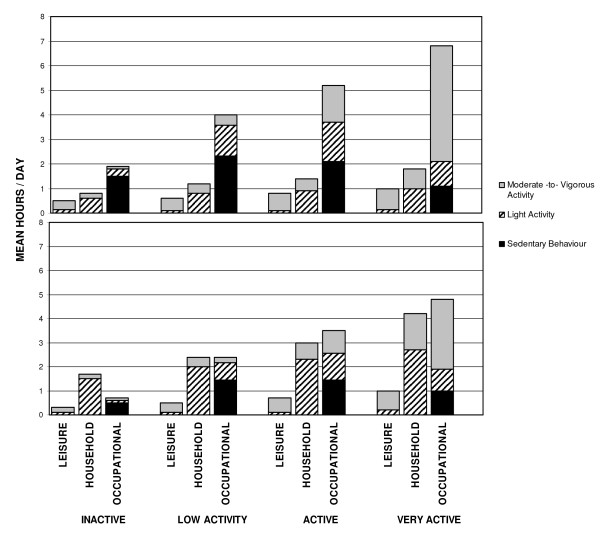
**Domain-specific hours and time spent sedentary and active**. Daily number of hours that men and women spend sedentary and in light and moderate-to-vigorous activity within domains of leisure, household work and occupation in the Tomorrow Project in Alberta Canada (2001 to 2005).

In both men and women, incremental decreases in total hours of activity from *very active *to *inactive *groups were accounted for by decreases in time spent in leisure-time, occupational and household-related activities (Figure 1) (Jonckheere-Terpstra (J-T) trend test: P<.0001). In men, however, the greatest differences in active time were in occupational hours of activity. In women, differences were seen in both occupational and household-related activities. Figure 1. illustrates the breakdown of time spent sedentary, and in light and moderate-to-vigorous activities within domains of activity. Among men, sedentary time and time spent in light and moderate-to-vigorous activities, varied across all activity levels, most noticeably in the occupational domain (J-T trend test for Sedentary and moderate-to-vigorous: P<.0001; Light: P = 0.0003). *Inactive *men were predominantly sedentary in their occupation. Time spent in moderate-to-vigorous activity increased with PAL with *very active *men spending the majority of their time in moderate-to-vigorous activity occupational activity (J-T trend test: P<.0001). Among women, differences in light and moderate-to-vigorous activities occurred in both household and occupational domains across PALs, with more noticeable increases observed in moderate-to-vigorous activity between *active *and *very active *levels.

### Activity-related energy expenditure in the different domains

Transportation-related energy expenditure was negligible (less than 0.5% of TEE) in both genders regardless of activity level.

Among men, activity-related energy expenditure ranged from 14% of TEE in the *inactive *group to 47% in the *very active *group. Occupation was associated with the highest activity-related energy expenditure in men classified as *very active *(32%), *active *(18%) and *low active *(11%) (J-T trend test P<.0001). Within each PAL stratum and across strata, the proportion of energy expenditure from leisure-time and household activities did not vary substantially for *very active, active *and *low active *men. Among men classified as *inactive*, occupational, household, and leisure-time activities were on average comparable in mean activity related energy expenditure contributions to total energy expenditure. Distributions of domain-specific percent contributions to TEE, however, were skewed to the right in this *inactive *stratum, indicating that the majority of men were completely sedentary (expending 1.5 METs/kg/hour or less), particularly in occupation (Table 2).

**Table 2:** Daily activity-related energy expenditure in domains as a proportion (mean [SD]; median (IQR)) of total energy expenditure ^ab^.

	Domains of activity									
	
	Men (N = 6,134)					Women (N = 9,457)				
**Activity**	**N**	**Leisure**	**Occupational**	**Household**	**All domains**	**N**	**Leisure**	**Occupational**	**Household**	**All domains**
**Very active (PAL≥1.9)**	3028	8.1 [7.7] 5.7 (10.1)	31.5 [12.4] 32.7 (16.8)	7.3 [6.4] 5.9 (7.0)	47.2 [6.0] 46.4 (9.3)	4062	7.6 [7.0] 5.6 (9.0)	20.8 [12.1] 21.3 (17.6)	17.5 [10.6] 14.9 (13.1)	46.1 [5.9] 44.9 (8.6)
										
**Active (1.6≤PAL<1.9)**	1840	8.2 [6.5] 6.9 (9.5)	17.6 [8.7] 17.6 (11.7)	7.3 [5.8] 5.9 (6.8)	33.4 [2.8] 33.5 (4.9)	3328	6.7 [5.4] 5.4 (7.3)	12.0 [8.1] 12.3 (13.0)	14.7 [7.3] 13.4 (9.8)	33.5 [2.8] 33.5 (4.8)
										
**Low activity (1.4≤PAL<1.6)**	919	6.6 [5.3] 5.4 (7.5)	10.9 [7.1] 11.8 (10.3)	6.9 [5.5] 5.4 (6.3)	24.6 [2.5] 24.9 (4.4)	1637	5.0 [4.1] 4.0 (5.6)	6.6 [6.0] 5.9 (11.1)	13.0 [5.8] 12.4 (8.5)	24.7 [2.5] 25.1 (4.2)
										
**Inactive (PAL<1.4)**	347	4.5 [4.2] 3.2 (5.8)	4.6 [5.3] 1.6 (9.5)	5.0 [4.1] 4.1 (5.5)	14.2 [4.9] 15.7 (5.9)	430	2.9 [2.8] 2.2 (3.9)	1.9 [3.4] 0.0 (2.2)	10.0 [4.9] 10.8 (7.6)	14.9 [4.6] 16.2 (5.2)
										
Kruskal-Wallis rank sum tests		P<.001	P<.001	P<.001			P<.001	P<.001	P<.001	

^a ^Includes people recruited in the first six recruitment waves (February 2001-January 2005) but excludes people who did not return a PYTPAQ (n = 2,405), pregnant women (n = 31), people who were recruited as 'second in household' in the first recruitment wav e (n = 344), people with prior history of cancer (n = 33), and people with missing data on body weight or height (n = 39).

^b ^Differences in median or distribution shape between activity levels for each domain were compared by using Kruskal-Wallis rank sum tests and were statistically significant (P<.001), Statistical significance of the Kruskal-Wallis rank sum test in each domain can be interpreted as follows: at least one median or distribution shape of the daily activity-related energy expenditure in a particular activity level significantly differed from another median or distribution shape in another activity level.

In men, the greatest difference in energy expenditure between *very active *and *inactive *groups was observed in the occupational domain. Occupational activity appeared to be the major determinant of PAL such that *very active *men expended on average 1000 kcal more per day than men classified as *low activity *and *inactive *(results not shown). In contrast, compared with men in *low activity *and *inactive *groups, *very active *men expended only about 100 to 150 kcal more per day in leisure-time and household activities, respectively.

Activity-related energy expenditure ranged from 15% of TEE in *inactive *women to 46% in *very active *women. Activity-related energy expenditure from occupational and household activities was comparable within *very active *and *active *categories (Table 2). In women in *low activity *and *inactive *groups, household activity was almost twice and five times the average percent of activity-related energy expenditure from occupational activity, respectively. The majority of *inactive *women, however, did not expend energy in occupation-related activity (median 0; IQR 2.2). Across PAL categories, the greatest differences in activity-related energy expenditure were seen in decreasing occupational activity from *very active *to *inactive *groups (J-T trend test P<.0001). The absolute average difference in occupational activity-related energy expenditure between *very active *and *inactive *women was almost 700 kcal per day (results not shown).

Owing to higher levels of activity-related energy expenditure from occupation (26% in men and 21% in women), fully employed men and women had slightly higher levels of overall activity-related energy expenditure compared with those working part-time or those classified as 'not employed/homemaker/student/other' (Table 3). Retired men and women had the lowest level of overall activity-related energy expenditure. Among retired men activity-related energy expenditure was greatest for leisure-time and household-related activity (11% and 12%, respectively), whereas among retired women it was greatest for household-related activity (19%).

**Table 3:** Daily activity-related energy expenditure as a proportion (mean [SD]; median (IQR)) of total energy expenditure by employment status^ab^.

	Domains of activities									
	
	Men (N = 6,131)					Women (N = 9,452)				
**Employment status**	**N**	**Leisure**	**Occupation**	**House**	**All domains**	**N**	**Leisure**	**Occupation**	**Household**	**All domains**
**Full-time**	4614	7.2 [6.4]	25.9 [12.2]	6.2 [4.8]	39.6 [10.4]	4123	6.4 [5.8]	20.7 [9.4]	11.7 [6.2]	39.0 [9.3]
		5.6 (8.7)	24.4 (19.2)	5.2 (5.9)	39.8 (15.0)		4.8 (7.2)	19.1 (13.2)	10.5 (7.3)	38.6 (13.1)
										
**Part-time**	382	7.4 [7.5]	20.1 [12.2]	7.7 [6.2]	35.5 [10.8]	2166	6.5 [5.7]	14.8 [9.4]	15.4 [7.7]	36.8 [9.6]
		5.4 (8.5)	18.3 (18.4)	6.2 (7.0)	35.0 (14.2)		5.0 (7.0)	13.3 (13.2)	13.8 (8.8)	36.5 (13.2)
										
**Not employed/homemaker/student/other**	372	7.2 [7.2]	19.7 [16.4]	8.8 [7.4]	35.9 [13.6]	1855	6.5 [6.1]	7.2 [10.7]	21.3 [10.7]	35.0 [11.6]
		5.0 (9.8)	17.7 (29.5)	7.2 (9.3)	37.1 (20.2)		4.9 (7.8)	1.9 (10.5)	19.6 (14.0)	34.6 (16.2)
										
**Retired**	763	10.8 [8.5]	6.5 [10.6]	11.6 [8.7]	28.9 [11.8]	1308	7.7 [6.7]	4.0 [7.3]	18.9 [8.7]	30.7 [10.4]
		9.1 (11.9)	0.9 (9.0)	9.6 (11.8)	28.4 (15.3)		5.9 (8.5)	0.7 (4.6)	17.7 (10.7)	30.0 (13.4)
Kruskal-Wallis rank sum tests		P<.001	P<.001	P<.001			P<.001	P<.001	P<.001	

^a ^Includes people recruited in the first six recruitment waves (February 2001-January 2005) but excludes people who did not return a PYTPAQ (n = 2,405), pregnant women (n = 31), people who were recruited as 'second in household' in the first recruitment wav e (n = 344), people with prior history of cancer (n = 33), and people with missing data on body weight or height (n = 39). Note: 3 men and 5 women had missing data on employment status.

^b ^Differences in median or distribution shape between employment status groups for each domain were compared by using Kruskal-Wallis rank sum tests and were statistically significant (P<.001), Statistical significance of the Kruskal-Wallis rank sum test in each domain can be interpreted as follows: at least one median or distribution shape of the daily activity-related energy expenditure in a particular employment status group significantly differed from another median or distribution shape in another employment status group.

## Discussion

In this cohort of Canadian adults reporting high levels of physical activity, we observed relatively low levels of leisure-time activity, compared with occupational and household activity. On average, these latter two domains accounted for more than 80% of overall hours of daily physical activity, the majority of daily activity-related energy expenditure, and accounted for differences in PAL categories among both men and women. Transportation-related physical activity (which could potentially make significant contributions to overall daily activity) was negligible in both men and women.

Among men, occupational activity appeared to be the most influential in determining activity level. The increases observed in time spent in light and moderate-to-vigorous activities within this domain, as levels of activity increased from *inactive *to *active*, suggest that all activities that are not sedentary play a role in determining activity level, with perhaps light activity being more important in preventing complete inactivity. Hence, among *inactive *groups, emphasis on even increasing levels of light activity (between 1.5 and 3 METs) may be helpful [28]. For *very active *men, less sedentary time and longer durations of moderate-to-vigorous activities were observed compared with men at lower levels of activity, suggesting that displacement of sedentary time may be necessary in order to achieve very high levels of activity. However, evidence linking sedentary behaviour to metabolic disorders and mortality risk, independent of overall activity, is sufficiently compelling to discourage sedentary behaviour [29-31].

Similarly, in women, occupational activity played a key role in determining activity level but household activity was also important. Statistics Canada has reported that women spend more time doing household chores than do men, even amongst co-habiting partners [32]. These findings are consistent with reports from other recent studies that have examined time spent in leisure-time, occupational, and household activities [19,33]. As also observed in men, participation in light activity appears to account for an increase in PAL at lower activity levels, while reductions in sedentary time and increases in moderate-to-vigorous activity play more prominent roles in achieving higher activity levels.

On average, *very active *men and women report about one hour per day in leisure-time activity which complies with most physical activity recommendations for chronic disease prevention [1,5]. We also observed that the difference in leisure-time activity between those who are *very active *and those who are *inactive *is only about 30 minutes in men and 40 minutes in women. In contrast, differences in hours of activity in occupational and household domains were much greater. This is predictable given that the majority of employed people spend much of their day at work and that household chores are usually engaged in on a daily basis. However, the majority of descriptive, etiologic and intervention studies have not focused on these domains. Probert et al [20] recently reported results suggesting that moderate-to-vigorous occupational physical activity was independently and more strongly associated with a lower risk of diabetes, heart disease and other chronic diseases than was leisure-time physical activity. Household and transportation-related activities were not studied.

Levine et al point out that the main component of TEE is non-exercise activity thermogenesis (NEAT), second only to basal metabolic rate and defined as energy expended above resting with the exclusion of formal exercise [34-36]. Therefore, emphasis on, and ascertainment of only leisure-time or structured activities may misrepresent overall activity level since leisure-time activity may not compensate for prolonged inactivity during the balance of the day. Indeed, *inactive *men and women engaged in about three hours of total activity per day from all domains and hence spent most of the day inactive. Had these individuals complied with physical activity recommendations to incorporate at least 30 minutes of moderate intensity aerobic activity on most days and an additional 20 minutes of vigorous-intensity activity on at least three days of the week they still would not have increased energy expenditure sufficiently to reclassify them to more active levels based on PAL [37]. It is also worth noting that leisure-time activity was highest amongst those who were retired, yet compared with other employment-status categories, retired men and women had lower levels of activity-related energy expenditure. A recent French study that examined physical activity and sedentary behaviour at retirement reported that while men and women increased leisure-time physical activity at retirement the increase did not compensate for the loss of occupational activity [38]. Hence, examining the unique activity patterns and lifestyle preferences of retired individuals as a subgroup independent of other employment status groups may be advisable in order to identify ways in which overall activity may be increased during this period of transition and major change in lifestyle.

A public-health concern is that those who are most inactive may become even more physically inactive and will soon spend even more time being sedentary as advances in technology continue to replace tasks of high energy expenditure with those requiring lower levels of energy expenditure [39]. Evidence is rapidly mounting to suggest that long periods of sitting time have adverse metabolic and health consequences that are not necessarily compensated for by shorter periods of discretionary leisure-time activity [30,39-42]. Hence, intervention studies are now underway to examine ways in which energy expenditure can be increased during working hours [43-45]. Traditionally, worksite physical activity programs have interrupted work schedules and lunch breaks in an effort to incorporate them into the day. Walking workstations [44] and office-place steppers [43] are not routinely accepted but interest and support for a more integrated 'be active while you work program' will undoubtedly increase as available and feasible options demonstrate their effectiveness [46].

Few studies have examined average time spent in transportation-related activity but health benefits have been reported for frequent commuting by foot and bicycle [47,48]. This domain could be targeted for messages that promote commuting by foot and bicycle particularly for those with inflexible work schedules and socioeconomic circumstances that make active forms of transportation more feasible to implement than leisure-time activity [18].

The strengths and limitations of this study must be considered. A clear strength of this study is the detail with which domain-specific activities were ascertained for time spent sedentary and in light and in moderate-to-vigorous activity. An additional strength is the geographically dispersed population-based sampling that was used to identify participants living in all regions of Alberta. While the response rate was estimated to be 32%, Bryant and colleagues have, compared the cohort with the Alberta component of the Canadian Community Health Survey (Statistics Canada) and observed that the populations were highly comparable on several sociodemographic characteristics [22]. In addition, we limit our analyses to comparisons of domains within and between PAL levels that are observed within this cohort. These methods maintain the internal validity of our results, despite also having excluded participants with missing data (physical activity, height and weight). Generalizability of these findings to other populations will need to be confirmed in future research.

Our findings rely on self-reported physical activity and while over-reporting of physical activity is likely present, these results reveal plausible and informative patterns of physical activity behavior that are generally consistent with our overall understanding of how people spend their time. Furthermore, the Canadian Health Measures Survey (CHMS) collected accelerometer measured physical activity data from a large sample of nationally representative men and women [49] and recently reported that on average two thirds of waking hours are spent sedentary (9.6 hours for men and 9.8 hours for women). Unfortunately, we did not measure sleep duration in this study since the PYTPAQ was designed to measure activity rather than inactivity. However, if one assumes on average 8 hours of sleep per day, then CHMS results are entirely consistent with our findings of on average a total of 8 hours of reported activity per day from all domains. In the CHMS, contextual information was not collected, precluding the exploration of activity and sedentary behavior across domains. The CHMS results, however, indicate that only 15% of adults meet target levels of 150 minutes per week of moderate-to-vigorous physical activity, further highlighting the critical need for a better understanding of activity patterns that could inform public health programs. In future studies it will be important to use both objective and self-reporting methods to better describe patterns of domain-specific activities that may provide insight into feasible and sustainable strategies that will increase activity and energy expenditure among those who spend most of their day inactive.

## Conclusion

We found that non leisure-time activities dominate the daily schedules of most people, and contribute the most to overall energy expenditure. The distribution of sedentary, light and moderate-to-vigorous intensity activities across domains is particularly informative and warrants further study. Enabling people to maximize their activity levels and energy expenditure from commonly performed daily activities across all domains of activity (particularly workplace and transport) may be the most feasible and sustainable approach to effectively increasing overall physical activity and health-related energy expenditure.

## Authors' contributions

IC conceptualized the study design and analysis and drafted the manuscript. GLS performed the statistical analysis, reviewed and edited the manuscript. CMF designed the Past Year Total Physical Activity Questionnaire, and reviewed and edited the manuscript. NO participated in drafting, reviewing and editing the manuscript. PJR participated in the design of the study, and reviewed and edited the manuscript. All authors have approved the final version.

## Competing interests

The authors declare that they have no competing interests.

## References

[B1] Warburton DE Katzmarzyk PT Rhodes RE Shephard RJ Evidence-informed physical activity guidelines for Canadian adults Can J Public Health 2007 98 Suppl 2 S16 S68 18213940

[B2] Warburton DER Nicol CW Bredin SSD Health benefits of physical activity: the evidence CMAJ 2006 174 801 809 10.1503/cmaj.051351 16534088 PMC1402378

[B3] World Cancer Research Fund, American Institute for Cancer Research Food, Nutrition, Physical Activity, and the Prevention of Cancer: A Global Perspective. Washington DC 2007

[B4] Warburton DER Nicol CW Bredin SSD Prescribing exercise as preventive therapy CMAJ 2006 174 961 974 10.1503/cmaj.1040750 16567757 PMC1405860

[B5] U.S.Department of Health and Human Services 2008 Physical Activity Guidelines for Americans 2008

[B6] Bruce MJ Katzmarzyk PT Canadian population trends in leisure-time physical activity levels, 1981-1998 Can J Appl Physiol 2002 27 681 690 10.1139/h02-040 12501004

[B7] Craig CL Russell S Cameron C Bauman A Twenty-year trends in physical activity among Canadian adults Can J Public Health 2004 95 59 63 14768744 10.1007/BF03403636 PMC6976221

[B8] Cameron C Craig CL Bull FC Bauman A Canada's physical activity guides: has their release had an impact? Can J Public Health 2007 98 Suppl 2 S161 S169 18213946

[B9] Juneau C Potvin L Trends in leisure-, transport-, and work-related physical activity in Canada 1994-2005 Prev Med 2010 51 384 386 10.1016/j.ypmed.2010.09.002 20832417

[B10] Kotz CM Levine JA Role of nonexercise activity thermogenesis (NEAT) in obesity Minn Med 2005 88 54 57 16475414

[B11] Katzmarzyk PT Tremblay MS Limitations of Canada's physical activity data: implications for monitoring trends Appl Physiol Nutr Metab 2007 32 S185 S194 10.1139/H07-113 18213948

[B12] Federal-Provincial and Territorial Advisory Committee on Fitness and Recreation Physical inactivity: a framework for action. Ottawa, ON 1997

[B13] Blair SN Lamonte MJ Nichaman MZ The evolution of physical activity recommendations: how much is enough? Am J Clin Nutr 2004 79 913S 920S 15113739 10.1093/ajcn/79.5.913S

[B14] Wareham NJ van Sluijs EM Ekelund U Physical activity and obesity prevention: a review of the current evidence Proc Nutr Soc 2005 64 229 247 10.1079/PNS2005423 15960868

[B15] Spanier PA Marshall SJ Faulkner GE Tackling the obesity pandemic: a call for sedentary behaviour research Can J Public Health 2006 97 255 257 16827420 10.1007/BF03405599 PMC6976261

[B16] Ford ES Li C Zhao G Pearson WS Tsai J Greenlund KJ Trends in low-risk lifestyle factors among adults in the United States: Findings from the Behavioral Risk Factor Surveillance System 1996-2007 Prev Med 2010 51 403 407 10.1016/j.ypmed.2010.08.002 20708637

[B17] Brown WJ Bauman AE Owen N Stand up, sit down, keep moving: turning circles in physical activity research? Br J Sports Med 2009 43 86 88 19001015 10.1136/bjsm.2008.055285

[B18] Berrigan D Troiano RP McNeel T Disogra C Ballard-Barbash R Active transportation increases adherence to activity recommendations Am J Prev Med 2006 31 210 216 10.1016/j.amepre.2006.04.007 16905031

[B19] Dong L Block G Mandel S Activities Contributing to Total Energy Expenditure in the United States: Results from the NHAPS Study Int J Behav Nutr Phys Act 2004 1 4 10.1186/1479-5868-1-4 15169563 PMC416566

[B20] Probert AW Tremblay MS Gorber SC Desk Potatoes: The importance of occupational physical activity on health Can J Public Health 2008 99 311 318 18767278 10.1007/BF03403762 PMC6976073

[B21] Autenrieth C Schneider A Doring A Meisinger C Herder C Koenig W Huber G Thorand B Association between different domains of physical activity and markers of inflammation Med Sci Sports Exerc 2009 41 1706 1713 10.1249/MSS.0b013e3181a15512 19657301

[B22] Bryant H Robson PJ Ullman R Friedenreich C Dawe U Population-based cohort development in Alberta, Canada: A feasibility study Chronic Dis Can 2006 27 55 63 16867239

[B23] Friedenreich CM Courneya KS Neilson HK Matthews CE Willis G Irwin M Troiano R Ballard-Barbash R Reliability and validity of the Past Year Total Physical Activity Questionnaire Am J Epidemiol 2006 163 959 970 10.1093/aje/kwj112 16524954

[B24] Ainsworth BE Haskell WL Leon AS Jacobs DR Montoye HJ Sallis JF Paffenbarger RS Compendium of physical activities: classification of energy costs of human physical activities Med Sci Sports Exerc 1993 25 71 80 10.1249/00005768-199301000-00011 8292105

[B25] Ainsworth BE Haskell WL Whitt MC Irwin ML Swartz AM Strath SJ O'Brien WL Bassett DR Schmitz KH Emplaincourt PO Compendium of physical activities: an update of activity codes and MET intensities Med Sci Sports Exerc 2000 32 S498 S504 10.1097/00005768-200009001-00009 10993420

[B26] Schofield WN Predicting basal metabolic rate, new standards and review of previous work Hum Nutr Clin Nutr 1985 39 Suppl 1 5 41 4044297

[B27] Food and Nutrition Board, Institute of Medicine Dietary Reference Intakes for Energy, Carbohydrate, Fiber, Fat, Fatty Acids, Cholesterol, Protein, and Amino Acids (Macronutrients) 2002 Washington, DC: The National Academies Press 10.1016/s0002-8223(02)90346-9 12449285

[B28] Owen N Healy GN Matthew CE Dunstan DW Too Much sitting: The population health science of sedentary behaviour Exerc Sport Sci Rev 2010 38 105 113 10.1097/JES.0b013e3181e373a2 20577058 PMC3404815

[B29] Hamilton MT Hamilton DG Zderic TW Role of low energy expenditure and sitting in obesity, metabolic syndrome, type 2 diabetes, and cardiovascular disease Diabetes 2007 56 2655 2667 10.2337/db07-0882 17827399

[B30] Owen N Bauman A Brown W Too much sitting: a novel and important predictor of chronic disease risk? Br J Sports Med 2009 43 81 83 19050003 10.1136/bjsm.2008.055269

[B31] Katzmarzyk PT Church TS Craig CL Bouchard C Sitting time and mortality from all causes, cardiovascular disease, and cancer Med Sci Sports Exerc 2009 41 998 1005 10.1249/MSS.0b013e3181930355 19346988

[B32] Marshall K Converging Gender Roles. (75-001-XIE) 2006 Ottawa, Statistics Canada

[B33] Ready AE Butcher JE Dear JB Fieldhouse P Harlos S Katz A Moffatt M Rodrigue M Schmalenberg J Gardiner PF Canada's physical activity guide recommendations are a low benchmark for Manitoba adults.(Survey) Appl Physiol Nutr Metab 2009 34 172 10.1139/H08-143 19370047

[B34] Levine JA Non-exercise activity thermogenesis Proc Nutr Soc 2003 62 667 679 10.1079/PNS2003281 14692603

[B35] Levine JA Kotz CM NEAT--non-exercise activity thermogenesis--egocentric & geocentric environmental factors vs. biological regulation Acta Physiol Scand 2005 184 309 318 10.1111/j.1365-201X.2005.01467.x 16026422

[B36] Levine JA Vander Weg MW Hill JO Klesges RC Non-exercise activity thermogenesis: the crouching tiger hidden dragon of societal weight gain Arterioscler Thromb Vasc Biol 2006 26 729 736 10.1161/01.ATV.0000205848.83210.73 16439708

[B37] Haskell WL Lee IM Pate RR Powell KE Blair SN Franklin BA Macera CA Heath GW Thompson PD Bauman A Physical activity and public health: updated recommendation for adults from the American College of Sports Medicine and the American Heart Association Med Sci Sports Exerc 2007 39 1423 1434 10.1249/mss.0b013e3180616b27 17762377

[B38] Touvier M Bertrais S Charreire H Vergnaud AC Hercberg S Oppert JM Changes in leisure-time physical activity and sedentary behaviour at retirement: a prospective study in middle-aged French subjects Int J Behav Nutr Phys Act 2010 7 14 10.1186/1479-5868-7-14 20181088 PMC2834610

[B39] Hamilton MT Hamilton DG Zderic TW The role of low energy expenditure and sitting on obesity, metabolic syndrome, Type 2 diabetes, and cardiovascular disease Diabetes 2007 56 2655 2667 10.2337/db07-0882 17827399

[B40] Hamilton MT Hamilton DG Zderic TW Exercise physiology versus inactivity physiology: an essential concept for understanding lipoprotein lipase regulation Exerc Sport Sci Rev 2004 32 161 166 15604935 10.1097/00003677-200410000-00007 PMC4312662

[B41] Perkins GM Owen A Kearney EM Swaine IL Biomarkers of cardiovascular disease risk in 40-65-year-old men performing recommended levels of physical activity, compared with sedentary men Br J Sports Med 2009 43 136 141 18308887 10.1136/bjsm.2007.044420

[B42] Katzmarzyk PT Church TS Craig CL Bouchard C Sitting Time and Mortality from All Causes, Cardiovascular Disease, and Cancer Med Sci Sports Exerc 2009 41 998 1005 10.1249/MSS.0b013e3181930355 19346988

[B43] McAlpine DA Manohar CU McCrady SK Hensrud D Levine JA An office-place stepping device to promote workplace physical activity Br J Sports Med 2007 41 903 907 10.1136/bjsm.2006.034900 17513333 PMC2658993

[B44] Thompson WG Foster RC Eide DS Levine JA Feasibility of a walking workstation to increase daily walking Br J Sports Med 2008 42 225 228 10.1136/bjsm.2007.039479 17717060

[B45] Chau JY der Ploeg HP van Uffelen JGZ Wong J Riphagen I Healy GN Gilson ND Dunstan DW Bauman AE Owen N Are workplace interventions to reduce sitting effective? A systematic review Prev Med 2010 51 352 356 10.1016/j.ypmed.2010.08.012 20801153

[B46] Carr LJ Walaska KA Marcus BH Feasibility of a portable pedal exercise machine for reducing sedentary time in the workplace Br J Sports Med10.1136/bjsm.2010.079574 21324889

[B47] Sugiyama T Merom D Reeves M Leslie E Owen N Habitual active transport moderates the association of TV viewing with body mass index J Phys Act Health 2010 7 11 16 20231750 10.1123/jpah.7.1.11

[B48] Matthews CE Jurj AL Shu XO Li HL Yang G Li Q Gao YT Zheng W Influence of exercise, walking, cycling, and overall nonexercise physical activity on mortality in Chinese women Am J Epidemiol 2007 165 1343 1350 10.1093/aje/kwm088 17478434

[B49] Colley RC Garriguet D Janssen I Craig CL Clarke J Tremblay MS Physical activity of Canadian adults: Accelerometer results from the 2007 to 2009 Canadian Health Measures Survey Health Reports 2011 22 1 8 21510585

